# A randomized, controlled noninferiority study of adjustable compression wraps compared with inelastic multilayer bandaging used in the intensive complex decongestive therapy of lower leg lymphedema

**DOI:** 10.1016/j.jvsv.2025.102214

**Published:** 2025-02-25

**Authors:** Anett Reisshauer, Emmanouil Tsatrafilis, Simone Kornappel, Doerte Huscher, Max E. Liebl

**Affiliations:** a Department of Physical Medicine, Charité – Universitätsmedizin Berlin, Corporate Member of Freie Universität Berlin and Humboldt-Universität zu Berlin, Berlin, Germany; b Institute of Legal Medicine and Forensic Sciences, Charité – Universitätsmedizin Berlin, Corporate Member of Freie Universität Berlin and Humboldt-Universität zu Berlin, Berlin, Germany; c Institute of Biometry and Clinical Epidemiology, Charité – Universitätsmedizin Berlin, Corporate Member of Freie Universität Berlin and Humboldt-Universität zu Berlin and, Berlin Institute of Health, Berlin, Germany

**Keywords:** Lymphedema, Adjustable compression wraps, Complex decongestive therapy, Compression therapy, Inelastic multilayer bandaging

## Abstract

**Objective:**

We evaluated the noninferiority of adjustable compression wraps compared with inelastic multilayer bandaging in reducing lower leg volume during intensive complex decongestive therapy in patients with bilateral, symmetrical lymphedema. Secondary outcomes included application time, patient comfort, and side effects.

**Methods:**

A prospective, randomized controlled noninferiority trial was conducted on 24 patients with stage II to III bilateral leg lymphedema during a 10-day intensive complex decongestive therapy. Each patient received adjustable compression wraps on a randomized side, while the contralateral leg was treated with inelastic multilayer bandages. The primary outcome was the difference in volume reduction, measured by perometer for lower leg volume, with a predefined noninferiority margin of 50 mL. Application time was measured. Subjective parameters were assessed with visual analogue scales (range, 0-100).

**Results:**

Adjustable compression wraps were noninferior to inelastic multilayer bandages with a mean difference in volume reduction of 9.45 mL (95% confidence interval −30.40 to 49.26), with respect to the a priori defined noninferiority margin of 50 mL. In a subsequent test of superiority, intraindividual differences did not differ significantly (*P* = .629). Adjustable compression wraps took significantly less time to apply (8.4 minutes; *P* < .001), and patients rated them as more comfortable (median visual analogue scale difference, 30; *P* < .001). Both compression methods reduced skin tightness significantly, with no notable differences between treatments. Adjustable compression wraps showed fewer side effects, and 83.3% of participants indicated they would continue using them. No adverse events were attributed to the compression wraps.

**Conclusions:**

Adjustable compression wraps proved to be an effective, noninferior alternative to inelastic multilayer bandages for reducing lower leg volume in the intensive complex decongestion therapy, although superiority could not be demonstrated. Their advantages include shorter application time and improved patient comfort. The potential for self-management makes them a promising option for lymphedema therapy, provided patients receive adequate training.

**Article Highlights**

• **Type of Research:** Single-center prospective randomized controlled noninferiority study
• **Key Findings:** Adjustable compression wraps were not inferior in terms of volume reduction compared with inelastic multilayer bandaging during the intensive complex decongestive therapy of 24 patients with bilateral lower leg lymphedema, with a mean difference in calf volume reduction of 9.46 mL (95% confidence interval, −30.40 to 49.26 mL), with an a priori defined noninferiority margin of 50 mL.
• **Take Home Message:** Adjustable compression wraps could be an alternative to inelastic multilayer bandaging in the intensive complex decongestive therapy of lymphedema.

Compression therapy plays a crucial role in the treatment of lymphedema.^1^ Several international guidelines and authors recommend complex decongestive therapy (CDT), of which compression therapy is an indispensable component in most cases.^2^, ^3^, ^4^, ^5^, ^6^, ^7^, ^8^, ^9^ In the initial, intensive phase of CDT (iCDT), compression is used primarily to reduce excess volume and normalize tissue homeostasis, usually administered together with manual lymphatic drainage, skin care, and exercise on a daily basis for several days to weeks. One very common way of compression is inelastic multilayer bandaging (IMB). This is followed by a maintenance phase (mCDT), in which the bandaging is replaced by custom-made compression garments with the aim of volume maintenance.^1^, ^2^, ^3^, ^4^, ^5^, ^6^, ^7^, ^8^, ^9^ Other authors recommend a three-phase approach with a longer optimization phase that lies between the intensive and maintenance phases.^10^

In recent years, the compression treatment of lymphedema has become more flexible with the introduction of medical adaptive compression wraps (ACWs).^1^ ACW are specially designed bandages with hook and loop fastening straps, which can be regularly readjusted individually, which is why they are referred to as adaptive. They are considered to be safe and easy to self-manage.^11^^,^^12^

Clinical data for ACW in lymphedema therapy are scarce, yet rather positive. The first study known to the authors dates back to 1996 and investigated the effect of ACW on volume reduction and venous hemodynamics.^13^ Damstra and Partsch^14^ showed that, in patients with moderate to severe lymphedema of the legs, ACW achieved a significantly greater volume reduction than IMB within 24 hours, whereby they attributed the effect mainly to individual pressure adjustments. Faerber et al^15^ observed a significant reduction in edema after 6 weeks of ACW therapy. After 3 weeks of intensive treatment, Borman et al^16^ found that ACW achieved a similar volume reduction as conventional multilayer bandages and improved quality of life and concluded that ACW can be a comfortable alternative to conventional multilayer bandages in iCDT.

In vivo pressure measurements allow a deeper understanding of the physiological effects of subcompression pressures. Reißhauer et al^17^ showed that the compression pressure in (unadjusted) ACW remains largely stable over time, after an initial pressure decrease owing to decongestive effects, as is known from IMB. The authors also demonstrated decreasing pressure gradients from distal to proximal measurement points.^17^ Partsch^18^ concluded that pressure measurements under self-applied ACW are reliable and appropriate. Reißhauer et al^19^ showed that ACW are not inferior to compression stockings in the mCDT for lymphedema.

In this study, we hypothesized that compression therapy with ACW would be noninferior to short-stretch bandaging in the iCDT. We used the reduction in lower leg volume as the primary outcome and defined a rigorous equivalence margin of 50 mL. If noninferiority was demonstrated successfully, a hierarchical test for superiority would follow. Subjective patient experience, application time, wear time, and clinical side effects were assessed as secondary end points.

## Methods

We conducted an investigator-initiated, single-center, prospective, controlled, randomized observational noninferiority study for patients with bilateral, largely symmetrical lower leg lymphedema (stages II and III) and an indication for iCDT. The selection of bilaterally affected patients allowed an intraindividual comparison of the volume effects with randomization of the intervention side. No changes were made to the study design after the trial commencement. The study was approved by the Ethics Committee of Charité Universitätsmedizin Berlin (EA4/161/22) and was registered in the German Registry of Clinical Studies (DRKS00030816) before the first patient was enrolled.

The patients were recruited at a specialized university lymphedema clinic. Patients were eligible for recruitment if they had a largely symmetrical (±170 mL lower leg volume difference), bilateral lymphedema stages II or III, and an indication for iCDT. Further inclusion criteria were the physical and mental capability to self-apply ACWs, as well as adult age (≥18 years). Exclusion criteria were circumference measurements that exceeded the ACW manufacturer's maximum specifications, skin folds with the need of additional pelotting, unclear cause of edema, and any contraindications to parts of the CDT. After informed consent was obtained, the patients underwent 10 days of iCDT in an outpatient setting.

For the time of the iCDT, the patients received manual lymphatic drainage and skin care before they received compression therapy on a daily basis. The randomized intervention side of each patient was treated with ACW (ACS Light, Juzo, Aichach, Germany), and the contralateral control side was treated with IMB. To avoid intraindividual differences, manual lymphatic drainage and IMB were applied by experienced physiotherapists with certified lymphedema specialization, and ACW were applied solely by the patients themselves. Skin care advice, disease-specific patient education, and exercise therapy or training advice were given to all study participants.

The segmental ACW components were selected individually, based on circumference measurements of the limb, and according to the manufacturer's instructions. Patients were thoroughly instructed in the correct handling of the self-application. They were instructed to wear both compression materials at least during the day, whereby the compression on both sides—intervention and control—should always be worn for the same length of time, and to readjust the compression pressure of the ACW at their own discretion. At night, both compression systems could be removed. A standardized clinical lymphological examination was performed before and after the intervention. After the study, all patients were supplied with individually measured compression stockings for the subsequent maintenance phase.

The primary outcome was the difference in volume effects achieved with the two compression systems during the iCDT. For reasons of comparability, only the lower leg volumes were measured, regardless of whether proximal edema was present or not. Lower leg volume was measured optoelectronically (Perometer 400 NT, Pero-System Messgeraete GmbH, Germany) below the tibial tuberosity, before the beginning of the iCDT (t0) and on day 10 (t1).^20^ Secondary outcomes were:

• application time (during the first physiotherapy session)
• wearing time (diary)
• frequency of readjustment of the ACW (diary)
• subjective perception (100 mm visual analogue scales)
  ○ wearing comfort (0 [extremely uncomfortable] to 100 [extremely comfortable])
  ○ feeling of tightness of the skin (0 [not at all] to 100 [very high])
  ○ overall handling of the ACW (0 [very easy] to 100 [very difficult])
  ○ handling of ACW application (0 [very easy] to 100 [very difficult])
  ○ handling of ACW removal (0 [very easy] to 100 [very difficult])
  ○ side effects such as itching, skin imprints, skin redness and bandaging slippage (patient diary).

At the end, the patients were asked about their willingness to continue using the ACW after the study. No changes were made to the methodology or the outcomes after the start of the study.

### Sample size estimation

On the basis of previous experience, we expected average volume reductions of 400 mL for the control treatment. For noninferiority testing, we a priori defined a rigorous equivalence threshold of 50 mL. The intraindividual difference in volume reduction between the intervention and control treatment was assumed to be on average −20 ± 76 mL, based on a pilot study where a mean difference of −15.7 and a mean:SD ratio of 3.8 was observed. For a one-tailed *t* test (α = 0.025) with 80% power, a noninferiority threshold of 50 mL and a conservative H0 difference of 0 with a standard deviation of 76, 21 patients were required. Assuming a drop-out rate of 15% owing to co-existing health risks during the iCDT, the sample size was calculated with 25 patients. Sample size estimation was performed using PASS software version 16.0.^21^ The randomization list was created with a Bernoulli distribution with probability 0.5 in IBM SPSS Statistics version 28 (IBM, Chicago, IL).

### Statistical analyses

As a primary outcome, the 95% confidence interval (CI) for the individual difference in volume reduction between intervention and control treatment was calculated and related to the a priori defined noninferiority threshold.^22^ Under the assumptions made, in case of noninferiority the estimated number of cases would also be sufficient to test hierarchically for clinically relevant superiority of the intervention over the control treatment of 50 mL or more in a one-sided paired *t* test (α = 0.025). Secondary outcome variables and other demographic and clinical characteristics were analyzed descriptively according to their scaling and distribution characteristics, and parametric (paired-samples *t* test) or nonparametric (Wilcoxon test, and McNemar test) tests were performed accordingly. The resulting *P* values are considered exploratory. Analyses were performed using IBM SPSS Statistics Version 29 (IBM).

The order of presentation follows the recommendations of the extension of the CONSORT statement for reporting noninferiority and equivalence trials.^22^

## Results

**Fig 1:**
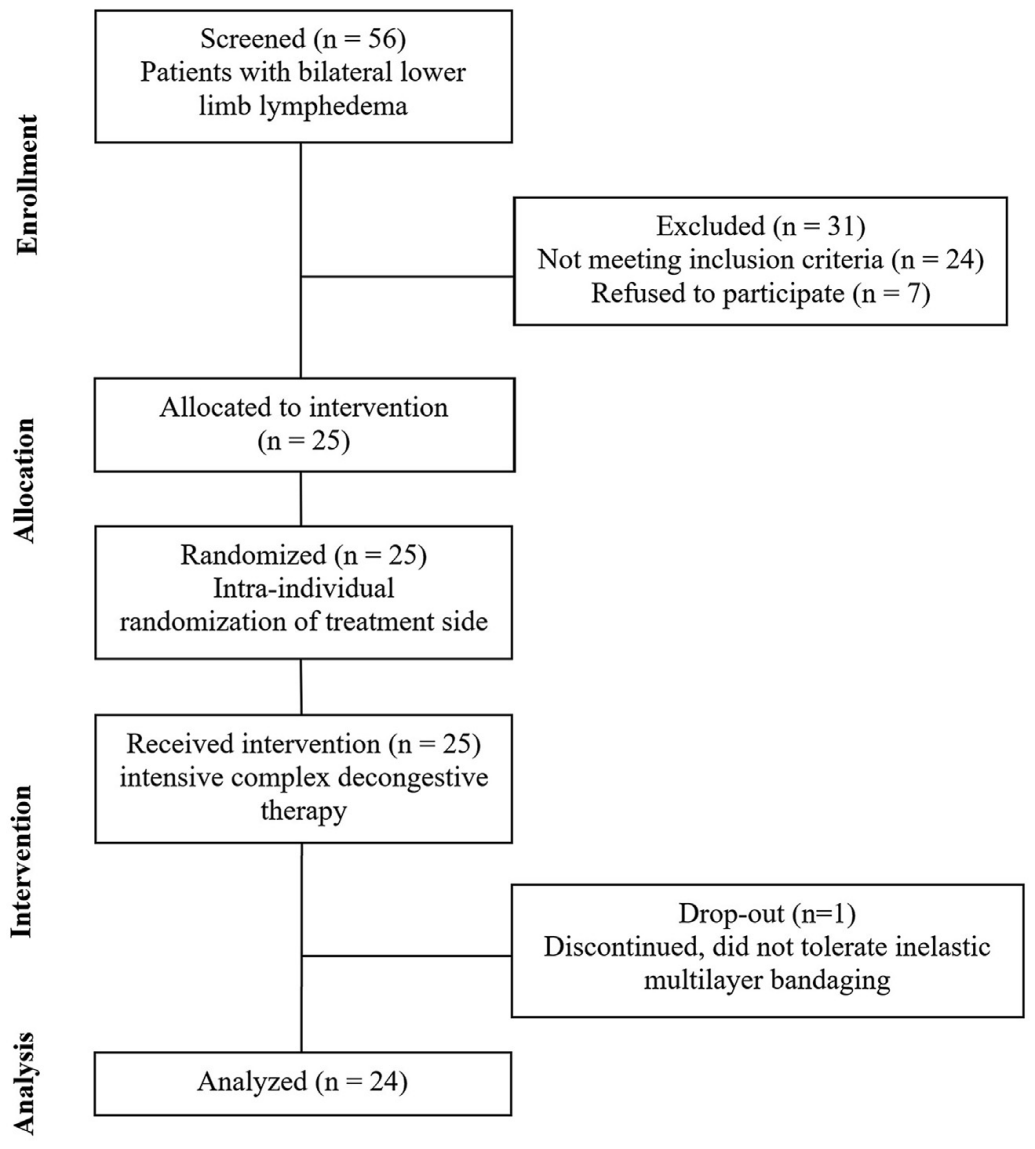
Flow chart.

Of the 25 recruited patients, 24 completed the iCDT and were analyzed for the primary outcome. There was one dropout (one patient did not tolerate the iCDT, in particular the IMB). Fig 1 shows the flowchart of the study. Recruitment and follow-up took place between November 2022 and July 2023. Table I provides an overview of the participants' characteristics.

**Table I:** Patient characteristics

Characteristics	Patients (n = 24)
Female sex	83.3 [20]
Age, years	62.5 ± 11.1; 31-79
Lymphedema	100 [24]
Primary lymphedema	58.3 [10]
Secondary lymphedema	41.7 [14]
With underlying malignant disease	8.3 [2]
With underlying CVI	8.3 [2]
With underlying obesity	8.3 [2]
More than one single cause	33.3 [8]
Duration, years	25.3 ± 13.9; 2-55
Weight, kg	91.0 ± 24.9; 51-158
Height, cm	165.4 ± 8.8; 153-184
BMI, kg/m^2^	33.1 ± 8.8; 22-59
Fibrosis^23^	
Mild	41.7 [14]
Moderate	58.3 [10]
Severe	0 [0]

*BMI,* Body mass index; *CVI,* chronic venous insufficiency.

Values are shown as a percentage [n] or as a mean value ± 8 standard deviation; minimum-maximum.

**Fig 2:**
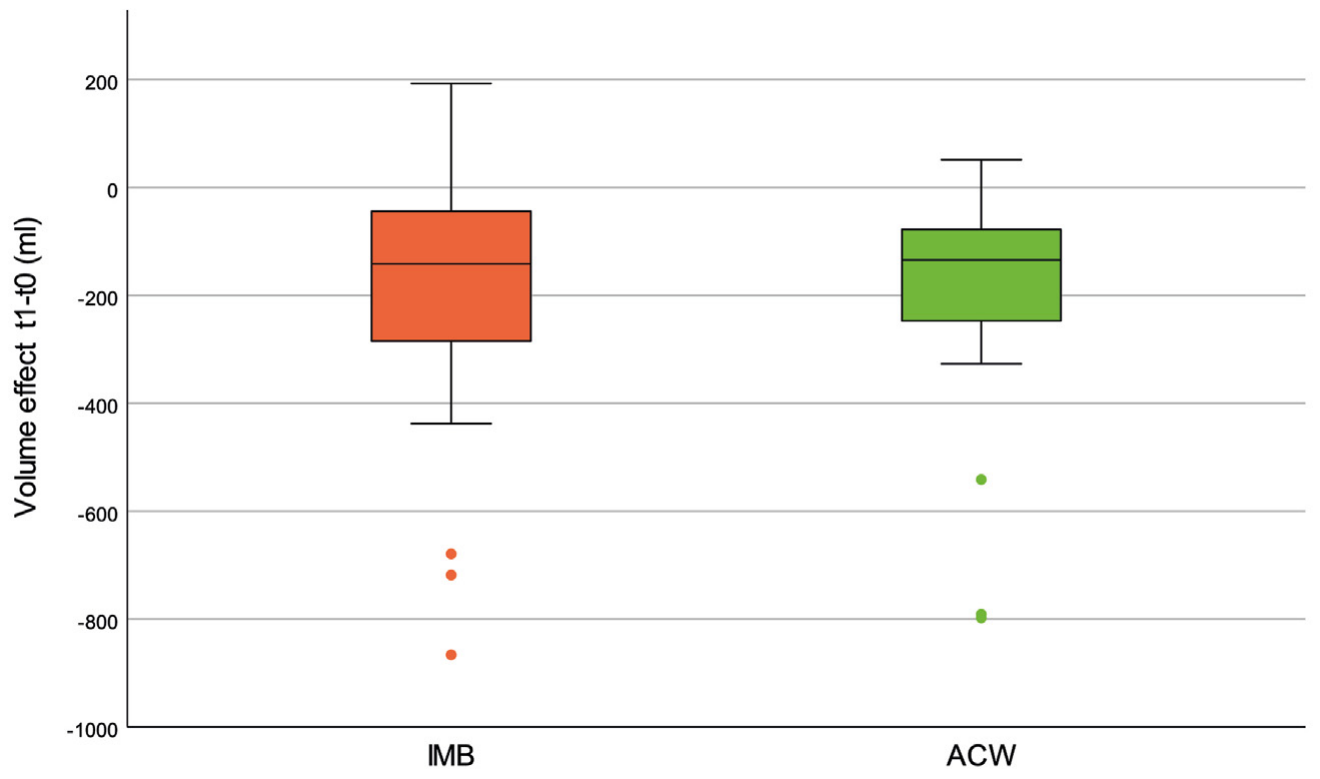
Box plot showing the volume effects of the adjustable compression wraps (*ACWs*) compared with the inelastic multilayer bandages (*IMBs*) before (t0) and after (t1) the intensive complex decongestive therapy (CDT).

**Fig 3:**
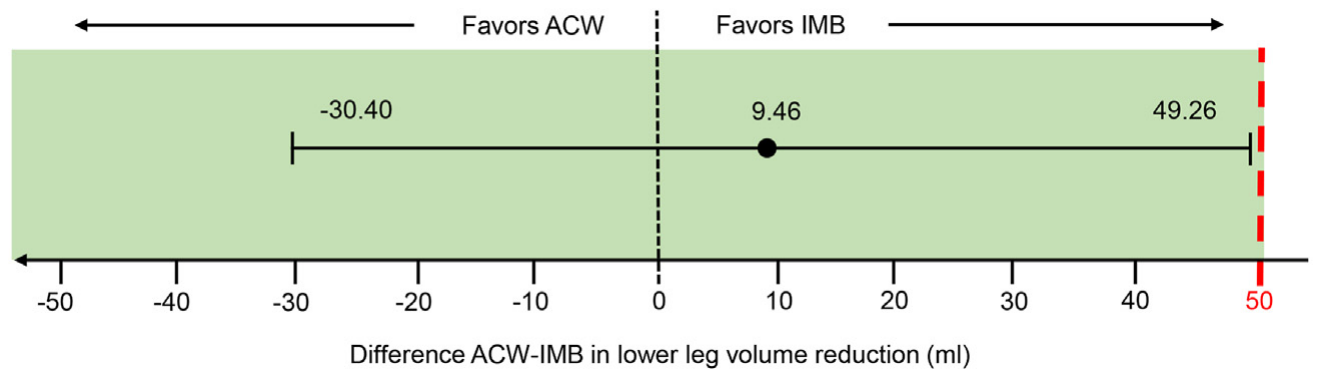
Diagram showing the difference between adjustable compression wraps (*ACWs*) and inelastic multilayer bandages (*IMBs*) before in lower leg reduction and 95% confidence interval (CI). The red dashed line indicates the noninferiority margin.

The volume measurements before (t0) and after (t1) iCDT are shown in Table II. Fig 2 shows the volume effects (ie the volume reduction achieved) of both treatments. The mean difference of the volume effects between ACW and IMB was 9.45 mL (95% CI, −30.40 to 49.26). Noninferiority of ACW was demonstrated by relating the CI to the a priori defined noninferiority threshold of 50 mL (Fig 3). The subsequent *t* test of the intraindividual differences was not significant (*P* = .629); therefore, no superiority could be shown.

**Table II:** Volume measurements of lower legs before and after the intervention

	Control (IMB)	Intervention (ACW)
Before iCDT	3500 (3128 to 4220)	3546 (3031 to 4210)
After iCDT	3320 (2865 to 5868)	3351 (2958 to 3976)
Δ Volume	−142 (−285 to −44)	−135 (−247 to −78)

*ACW,* Adaptive compression wraps; *iCDT,* intensive phase of complex decongestive therapy; *IMB,* inelastic multilayer bandaging.

Values are shown as median (interquartile range).

**Fig 4:**
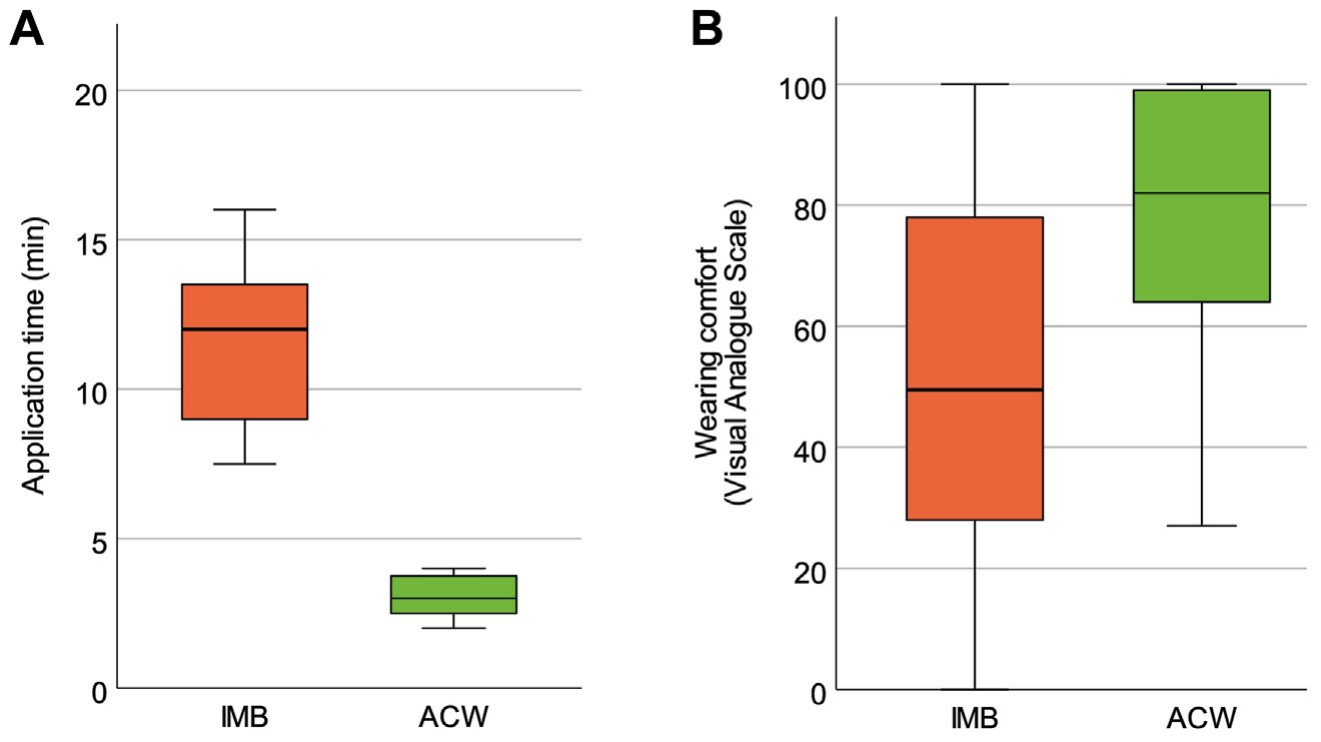
**(A)** Comparison of application times (minutes). **(B)** Comparison of wearing comfort (visual analogue scale from 0 [extremely uncomfortable] to 100 [extremely comfortable]). *ACW*, adjustable compression wrap; *IMB*, inelastic multilayer bandage.

The application time of the ACW was 3.0 ± 0.7 minutes, whereas IMB application with 11.5 ± 2.7 minutes needed significantly longer (*P* < .001) (Fig 4, *A*). Wear time was recorded for IMB with on average of 19.8 hours/day, similar to ACW with 20.9 hours/day. A majority of the probands (54.2%) readjusted the ACW pressure during the wearing time: 37.5% readjusted one to three times and 16.7% more than three times a day. Wearing comfort of ACW proved to be superior with a median visual analogue scale difference of 30 mm (interquartile range [IQR], 3-44 mm; *P* = .001) compared with IMB (Fig 4, *B*).

The feeling of skin tightness, a common lymphedema symptom, was surveyed separately for both sides before and after iCDT. Both types of compression were able to adequately reduce the feeling of tightness during the course of iCDT. On the intervention side and on the control side, the reduction was statistically significant (median ACW: −13.0 mm [IQR, −38 to 40 mm; *P* = .017]; IMB: −14.5 mm [IQR, −34 to 35 mm; *P* = .027]). Symptom reduction did not differ between the two treatments (median: 0.0 [IQR, −4.5 to 5.0; *P* = .903]).

Overall, the handling of ACW was perceived rather easy with a median visual analogue scale value of 20 mm, although its application (18 mm) was considered slightly more difficult than the removal (8 mm) (Supplementary Fig, online only).

With only 4 men in the study (difference of volume effects: range, 38-234 mL) our ability to evaluate gender differences was limited. In women (n = 20), the mean difference of the volume effects was −9.04 mL (95% CI, −48.95 to 30.88), favoring ACW, and did not differ remarkably when further subdividing by body mass index (BMI) (BMI < 30, n = 9: −12.67 mL [95% CI, −61.18 to 35.84]; BMI ≥ 30, n = 11: −6.06 mL [95% CI, −75.26 to 63.13]).

Side effects reported at t1 included itching, skin imprints, skin redness, and slipping. Although itching occurred with a similar frequency in ACW and IMB (20.8% vs 25.0%; *P* = .564), a significantly lower frequency of skin imprints (4.2% vs 41.6%; *P* = .007) was reported on the ACW side compared with the IMB side. Skin redness occurred less frequently on the ACW side (12.5% vs 29.2%; *P* = .157), as well as slipping of compression materials (20.8% vs 37.5%; *P* = .317). Twenty of 24 patients (83.3%) stated that they would continue to use the ACW after the study; 4 (16.6%) said they would not.

There were no adverse events in relation to ACW. The study dropout occurred owing to intolerance of compression bandaging.

## Discussion

This study demonstrated that adjustable compression bandages were noninferior to inelastic multilayer bandages in terms of volume reduction. In this respect, our study is consistent with other studies showing that ACWs are comparable with IMBs during iCDT in lower limb lymphedema^16^ or upper limb lymphedema.^24^ Damstra and Partsch^14^ and Mosti et al^25^ found ACW to be more effective than IMB in reducing lymphedema volume. In studies where ACWs were used as the sole lymphedema therapy—without manual lymphatic drainage—ACWs showed a statistically significant volume reduction in lower limb^15^ and upper limb lymphedema.^26^

Time is an important factor in clinical practice. In terms of time required for application, ACW was on average 8.4 minutes less time consuming than IMB. In addition, Faerber et al^15^ observed that the time required by patients for ACW application decreases over time. This allows more time for manual lymphatic drainage, as well as for exercise and skin care, which can bring significant benefits to the patient. Improved time management could also have a positive impact on individual self-management approaches to decongestive treatment.

A majority of the patients readjusted the ACW at least once per day. This finding supports the notion that the readjustment of the ACW, in particular, is crucial to reach therapeutic effects comparable with those of conventional bandaging methods, thereby promoting self-management and optimizing treatment effects.^12^^,^^14^^,^^18^^,^^25^^,^^27^

Asked about their perception of wearing comfort, patients rated ACWs as significantly more comfortable than IMBs, which confirms other studies.^15^^,^^16^^,^^26^^,^^27^ Mosti et al^25^ examined the initial decongestion phase in patients with phlebo-lymphedema and reported wearing comfort of ACW to be similar to IMB. The positive evaluation of the overall management, application, and removal of ACW is consistent with the findings of Damstra and Partsch,^14^ who found that autonomous management of ACW seems to improve clinical outcomes and represents a promising step towards self-management. Campanholi et al^26^ found ACW to be more practical than the compression bandage, whereas Dissemond et al^11^ refer to ACW as an easy-to-use treatment option in the management of patients with chronic leg ulcers and edema. The majority of respondents (83.3%) stated that they would continue using the ACW after the study, which is also in line with the results of another study.^15^

The absence of serious adverse events on the intervention side supports the safety of ACW, which was also the conclusion of previous ACW studies.^11^^,^^28^^,^^29^ The low side effect rates of ACW do not seem to be solely dependent on the material, but should be interpreted in conjunction with good patient education and training.^12^^,^^29^ Higher complication rates reported in one study seem likely to be related to application or instruction difficulties.^30^

From a clinical perspective, ACW could be beneficial, and from an economic perspective it could lead to cost savings. The financial impact of the treatment and the materials used in this study have not been quantified, but such calculations have already been performed by other researchers.^31^ The British National Institute for Health and Care Excellence states that ACW could be an option for treating patients with venous ulcers because they can decrease the cost of care.^32^ Mosti et al^27^ showed that ACW as a self-applied treatment solution is significantly cheaper and more effective, making it a cost-effective alternative to inelastic bandages in the treatment of venous leg ulcers. Dissemond et al^33^ also suggest there are indications for patient groups where the use of ACW could decrease the need for nursing care, thereby promoting patient independence. According to Ehmann et al,^34^ the use of ACWs could have the potential to decrease the overall burden on the health care system by decreasing the number of necessary specialist visits and costs, while still achieving good clinical outcomes.

ACWs have been recommended for patients with comorbidities associated with lymphedema, such as obesity, musculoskeletal conditions,^35^ and immobility, as well as for patients dependent on nursing care.^36^^,^^37^ In contrast, Balet et al^38^ identified comorbidities such as obesity, gripping difficulties, cognitive impairment, and low social status as factors that could limit the daily self-management of ACWs. A clear indication, taking into account contraindications and risks, should, therefore, be considered just as important as the careful instruction in its use.

Given the positive patient acceptance with regard to application and tolerability, ACWs could be useful in any situation with a volume exacerbation, eg during maintenance phase, in prolonged immobility, or exposure to warm weather conditions like heat days. It may be also useful in the interval between iCDT and mCDT, until individualized compression hosiery is available. Additionally, other authors have proposed that ACWs could be regarded as the sole therapeutic option in the absence of iCDT modalities,^39^ like when residing in a remote area.^26^

This study has potential limitations. We chose a side-randomized design with bilateral symmetrical lymphedema to reduce systematic errors. This design increased comparability, but could also lead to selection bias, because patients with stage III lymphedema were actually excluded; in practice, symmetrical stage III lymphedema with a given symmetry in such narrow limits of side difference is rare. In addition, the product's maximum garment sizes for patients with excessive edema represent a further limitation. Compared with the average lower leg volume of 3750 mL at t0, our a priori defined threshold for noninferiority of 50 mL seems to be rigorously chosen, with a proportion of 1.3% even lower than the usual volume variation during a day. As close as the results of the study may seem, in this context the overall result seems all the clearer. Owing to the small number of men included, we were unable to analyze gender differences, but the numbers suggest possible inequalities that should be investigated. Another limitation is the volume measurement method used, because the used perometer has a technical limit of the distal measurement at 53 mm.^40^ The scalability of our results is limited owing to the fact that different ACW manufacturers use different material. Its stiffness—an important parameter in regard of decongestion—can differ significantly between products. The stiffness of most ACWs seems to have rather short-stretch properties. However, health professionals should be aware of the ACW's properties they prescribe or recommend, according to the compression class in hosiery.

## Conclusions

Adjustable compression wraps can be a compelling alternative or complementary option in the iCDT for lymphedema. The successful integration of ACWs into clinical practice depends on thorough training of patients in the application technique to ensure both therapeutic efficacy and safety.

## Author Contributions

Conception and design: AR, DH, ML

Analysis and interpretation: AR, ET, DH, ML

Data collection: ET, SK, ML

Writing the article: AR, ET, ML

Critical revision of the article: AR, ET, SK, DH, ML

Final approval of the article: AR, ET, SK, DH, ML

Statistical analysis: DH

Obtained funding: AR, ML

Overall responsibility: AR

AR and ET contributed equally to this article and share co-first authorship.

## Funding

Funded by Juzo (Julius Zorn GmbH, Aichach, Germany). The funding source did not influence study design, collection, analysis, or interpretation of data, manuscript writing, creation of the manuscript, or the decision to submit the manuscript for publication.

## Disclosures

A.R. received research funding and honoraria for lectures from Juzo (Julius Zorn, Germany). E.T. received honoraria for lectures from Juzo (Julius Zorn, Germany). S.K. received honoraria for lectures from Juzo (Julius Zorn, Germany). M.L. received honoraria for lectures from Juzo (Julius Zorn, Germany) and received research funding and honoraria by L&R (Lohmann & Rauscher, Germany).

## Appendix (online only)

Supplementary Fig Subjective patient experience of overall handling, application and removal of adjustable compression wraps (visual analogue scale from 0 [extremely uncomfortable] to 100 [extremely comfortable]). *ACW*, adjustable compression wrap; *IMB*, inelastic multilayer bandage.
