# Supplementary figures and images for: A randomized, controlled noninferiority study of adjustable compression wraps compared with inelastic multilayer bandaging used in the intensive complex decongestive therapy of lower leg lymphedema
Source: J Vasc Surg Venous Lymphat Disord. 2025 Feb 25;13(4):102214. doi: 10.1016/j.jvsv.2025.102214 (PMC11995056; doi:10.1016/j.jvsv.2025.102214)

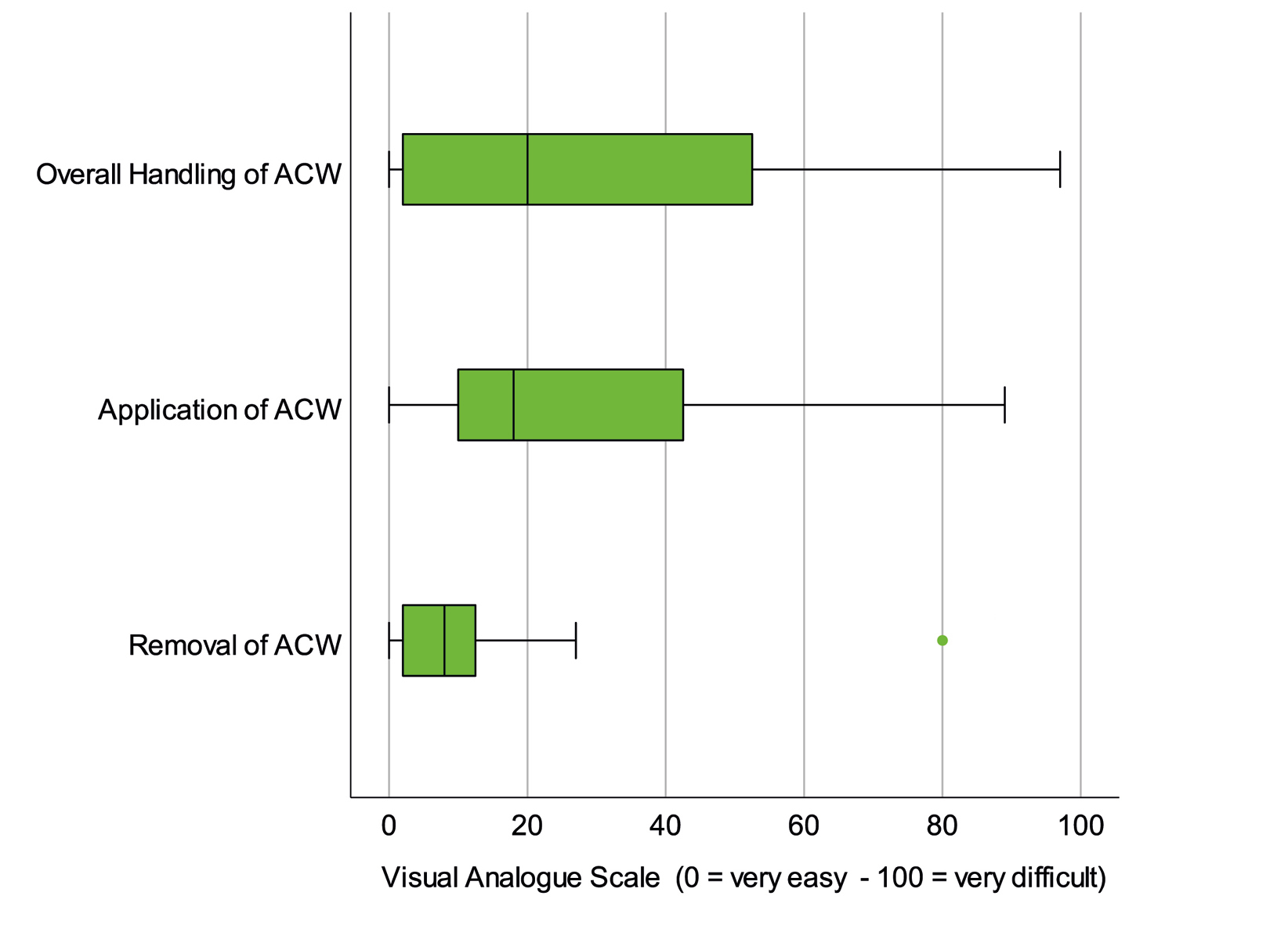

Supplement: Supplementary Fig — Subjective patient experience of overall handling, application and removal of adjustable compression wraps (visual analogue scale from 0 [extremely uncomfortable] to 100 [extremely comfortable]). ACW, adjustable compression wrap; IMB, inelastic multilayer bandage. [file figs1.jpg]
